# SETDB1 promotes progression through upregulation of SF3B4 expression and regulates the immunity in ovarian cancer

**DOI:** 10.1186/s13048-024-01358-8

**Published:** 2024-02-05

**Authors:** Hongjuan Yang, Lei Sui, Cuicui Cai, Huijun Chu, Yuchao Diao

**Affiliations:** 1 Department of Obstetrics and Gynecology, the Affiliated Hospital of Qingdao University, 16 Jiangsu Road, Qingdao, 266000 Shandong Province China; 2 Department of Gynecological Oncology, Qingdao Central Hospital, University of Health and Rehabilitation Sciences, Qingdao, 266000 Shandong China

**Keywords:** SETDB1, Transcription factor, SF3B4, Ovarian cancer, Tumour immunity

## Abstract

**Background:**

Ovarian cancer (OC) is the most lethal gynecologic malignant tumour. The mechanism promoting OC initiation and progression remains unclear. SET domain bifurcated histone lysine methyltransferase 1(SETDB1) acts as an oncogene in a variety of tumours. This study aims to explore the role of SETDB1 in OC.

**Methods:**

GEO, TCGA, CSIOVDB and CPTAC databases jointly analysed SETDB1 mRNA and protein expression. Effect of SETDB1 expression on the clinical prognosis of OC patients was analysed through online Kaplan‒Meier plotter and CSIOVDB database. Then, the effect of SETDB1 in OC cells progression and mobility was examined using MTT, EdU, colony formation and transwell assay. Additionally, Cistrome DB database was used to visualize the binding of SETDB1 protein and splicing factor 3b subunit 4 (SF3B4) promoter, and dual-luciferase reporter gene assay was performed to confirm the interaction. Finally, bioinformatics analysis was employed to reveal the relationship between SETDB1 and the microenvironment of OC.

**Results:**

In the present study, we found that SETDB1 was obviously upregulated in OC and its overexpression predicted poor prognosis of OC patients. Then, we verified that SETDB1 promoted the progression and motility of OC cells in vitro. Knockdown of SETDB1 had the opposite effect. Further research showed that SETDB1 acted as a transcription factor to activate SF3B4 expression. SF3B4 knockdown impaired the effect of SETDB1 to promote the proliferative capacity and motility of OC cells. Finally, the results of bioinformatics analysis confirmed that SETDB1 regulated the immune microenvironment of ovarian cancer.

**Conclusion:**

SETDB1 promoted ovarian cancer progression by upregulating the expression of SF3B4 and inhibiting the tumour immunity. SETDB1 may be a promising prognostic and therapeutic marker for OC.

## Background

Worldwide, the number of new cases was 310,000 and the number of new deaths was 210,000 from OC in 2020 [1]. Both morbidity and mortality rank eighth among female malignant tumours [1]. OC patients have poor prognosis, and the reason for this is because of most patients are diagnosed in advanced clinical stages [2]. The rate of 5-year survival is approximately 30% in advanced ovarian cancer with extra-pelvic metastasis [3]. More than 70% of serous ovarian cancer patients develop drug resistance and relapse due to the complex of its clinicopathological features and molecular mechanism [4, 5]. Therefore, the mechanism promoting ovarian cancer progression remains to be explored.

SETDB1 is widely expressed in a variety of human tissues and results in gene silencing or transcriptional suppression by highly specific methylation of the histone 3 lysine 9 (H3K9) residue [6]. Less commonly, this molecule also acts as a transcription factor to promote gene expression [6, 7]. In humans, the SETDB1 gene is located on chromosome 1q21.3, and its length is 38.6 kb. This gene encodes the SETDB1 protein, which is made up of 1291 amino acids [8]. Several recent studies have confirmed that SETDB1 plays as an oncogene and widely amplified and overexpressed in human tumours [9]. These cancers include bladder cancer [10], breast cancer [11, 12], colorectal cancer [13], lung cancer [14, 15], and pancreatic ductal adenocarcinoma [16].

SETDB1 promotes tumour progression through multiple mechanisms, including gene silencing [16, 17], epithelial-mesenchymal transition (EMT) [18], drug resistance [12, 19], methylation and activation of AKT [20, 21], immune cell function and tumour immunotherapy [22, 23]. Other studies have shown that SETDB1 can also act as a gene transcriptional activator [24, 25]. However, SETDB1 has not been well studied in gynaecological tumours. Proteogenomic characterization of endometrial cancer identified that the SETDB1 protein levels were negatively correlated with the apoptotic protein TNFRSF 10B and tumour suppressor CDKN1A/p21 [26]. In ovarian cancer, serum circSETDB1 levels are a biomarker for chemotherapy resistance [27]. Knockdown of CircSETDB1 significantly decreased MAP3K3 expression in a miR-129-3p-dependent mechanism and inhibited serous ovarian cancer progression [28]. However, the exact mechanism of SETDB1 in ovarian cancer progression remains to be further researched.

RNA-binding proteins (RBPs), also known as splicing factors, are participated in the compose of spliceosomes and involved in post-transcriptional regulation [29]. Human RBPs account for approximately 7.5% of human protein-coding genes and involve in regulating gene expression [30, 31]. Many researches have shown that RBPs can regulate the biological behavior of multiple tumours [32–34]. SF3B4 is a core subunit of the splicing factor 3b (SF3B) complex, and an imbalance of SF3B4 involved in the occurrence of many diseases, such as Nager syndrome [35] and tumours [36, 37]. Our preliminary research shows that SF3B4 plays an oncogene role in gynaecological cancers [38, 39].

Results of the present study confirmed that SETDB1 expression was upregulated in ovarian cancer and its high expression predicted poor prognosis. Next, we demonstrated that SETDB1 promoted OC cell progression in vitro. Further study showed that SETDB1 promoted the transcription of SF3B4. Above findings suggest that SETDB1 may be a prognostic biomarker and potential therapeutic target in OC.

## Materials and methods

### Cell lines and cell culture

HEY and HEK293T cells were cultured in DMEM containing 10% fetal bovine serum (FBS). A2780 cells were cultured in RPMI 1640 plus 10% FBS. SKOV3 cells were cultured in McCoy’s 5 A medium containing 10% FBS. All the cells were authenticated for short tandem repeat (STR) profiling.

### RNA interference

SETDB1- and SF3B4-targeted small/short interfering RNA (siRNA) were purchased from GenePharma (Shanghai, China). We transfected siRNAs into OC cell lines using Lipofectamine 2000 (Invitrogen, 11,668–019). We extracted RNA samples one day after transfection and protein samples two days after transfection. The sequences of siRNA are listed in Table 1.

**Table 1:** si-RNA sequence used in this study

Method	Name	Sequence (5’-3’)
si-RNA	negative control	UUCUCCGAACGUGUCACGUTT
si-RNA	si-SETDB1#1	TCAGTGGCCGGCAAATGGG
si-RNA	si-SETDB1#2	TTAGTTCTCCAGACATCTG
si-RNA	si-SF3B4#1	GGAUGAGAAGGUUAGUGAATT
si-RNA	si-SF3B4#2	GCACCAAGGCUAUGGCUUUTT

### Plasmid construction and lentiviral infection

Open reading frames (ORFs) of SETDB1 in the GV492 vector were purchased from GeneChem (Shanghai, China). pMD2.G (Addgene, 12,259) and psPAX2 (Addgene, 12,260) were used for lentivirus packaging in HEK293T cells. HEY cell lines with SETDB1 stable overexpression were constructed by lentivirus infecting for 24 h and then puromycin (2 µg/ml) screening for 5–7 days.

### RNA extraction and quantitative real-time PCR (qRT–PCR)

TRIzol (Invitrogen, 15,596,018) reagent was used to extract RNA. cDNA was obtained using PrimeScript RT master mix kits (TaKaRa, RR037A). qRT‒PCR was performed using SYBR Green qPCR master mix (TaKaRa, RR420A). GAPDH was the endogenous control. The primer sequences are listed in Table 2.

**Table 2:** Primer sequence used in this study

Method	Name	Sequence (5’-3’)
qPCR	GAPDH-F	GGTCTCCTCTGACTTCAACA
qPCR	GAPDH-R	GTGAGGGTCTCTCTCTTCCT
qPCR	SETDB1-F	CAGCATGCGAATTCTGGGC
qPCR	SETDB1-R	CAGCAGGAGGGTGGTAATCA
qPCR	SF3B4-F	AGTCAACACCCACATGCCAA
qPCR	SF3B4-R	CACCCGTATTGGCTTCCCAT

### Protein extraction and western blotting (WB)

We extracted protein lysates using RIPA buffer (Beyotime Bio, P0013) with PMSF and NaF (100:1:1). We determined protein concentration using a BCA Protein Assay Kit (Merck Millipore, 71,287). WB was performed with specific antibodies against SETDB1 (1:1000, Proteintech, 11,231–1-AP), SF3B4 (1:8000, Proteintech, 10,482–1-AP), and β-actin (1:8000, Sigma-Aldrich, SAB3500350).

### MTT assay

One thousand cells per well were seeded into 96-well plates after transfection with siRNA for 24 h and cultured for 5 days. MTT (Sigma-Aldrich, 0.5 mg/ml) was added to the wells and incubated for 4 h at 37 °C. DMSO (Sigma-Aldrich, 100 µl) was added after the supernatant was removed. The absorbance at 450 nm was measured to draw a growth curve.

### EdU assay

EdU cell proliferation assay (EdU) was performed using an EdU Kit (Beyotime, C0071s). 15,000 cells per well were seeded into 96-well plates after transfection with siRNA for 24–48 h. The next day, EdU reagent (1:1000 dilution) was added to each well. Two hours later, the cells were fixed and stained.

### Migration and invasion assays

Migration and invasion assays were performed after transfection with siRNA for 24 h. In the upper chamber, we seeded 1 × 10^5^ cells with serum-free medium. In the lower chamber, we added medium with 20% FBS. Then, the cells were cultured for 6–48 h. Next, the cells were fixed and stained, and migrated or invaded cells were counted.

### Colony formation assay

After siRNA transfection for 24 h, 800 cells per well were seeded into 6-well plates and cultured for approximately 10 days with appropriate culture conditions. Then, the medium was changed and the cell growth was observed regularly. Finally, the number of cell clones was counted.

### Dual-luciferase reporter assay

The full-length sequence of SF3B4 promoter was cloned into pGL4.26 plasmids. Then, The SF3B4 promoter vector and SETDB1 siRNA were transfected into HEY cells or stable SETDB1-overexpressing HEY cells. The luciferase activity was measured using a dual-luciferase reporter assay system (Promega, E2920) 48 h after transfection.

### Bioinformatics analysis

SETDB1 mRNA and protein differential expression between OC and normal tissues was from the GEO database (https://www.ncbi.nlm.nih.gov/geo/), TCGA database, CSIOVDB database (http://csiovdb.mc.ntu.edu.tw/CSIOVDB.html) and CPTAC database (https://proteomics.cancer.gov/programs/cptac). The effect of SETDB1 expression on the clinical prognosis of OC patients was analysed through the online Kaplan‒Meier plotter (https://kmplot.com/analysis/) and CSIOVDB database. Correlation analysis between SETDB1 and SF3B4 mRNA expression was obtained from TCGA database. The Cistrome DB database (http://cistrome.org/db/) was used to visualize the binding of the SETDB1 protein and SF3B4 promoter. The MEXPRESS database (http://mexpress.be/) showed amplified methylation of SETDB1 in ovarian cancer. Correlation analysis between SETDB1 and immune cells abundance was analysed from the TISIDB database (http://cis.hku.hk/TISIDB/).

### Statistical analysis

In this study, all experiments were repeated three times. Student’s t test was used to determine statistical significance. Statistical analysis was performed using SPSS 25.0 software. The results are presented as the mean ± SD. *p* < 0.05 was considered statistically significant (*p < 0.05, **p < 0.01).

## Results

### SETDB1 is highly expressed in ovarian cancer and associated with poor prognosis

**Fig. 1:**
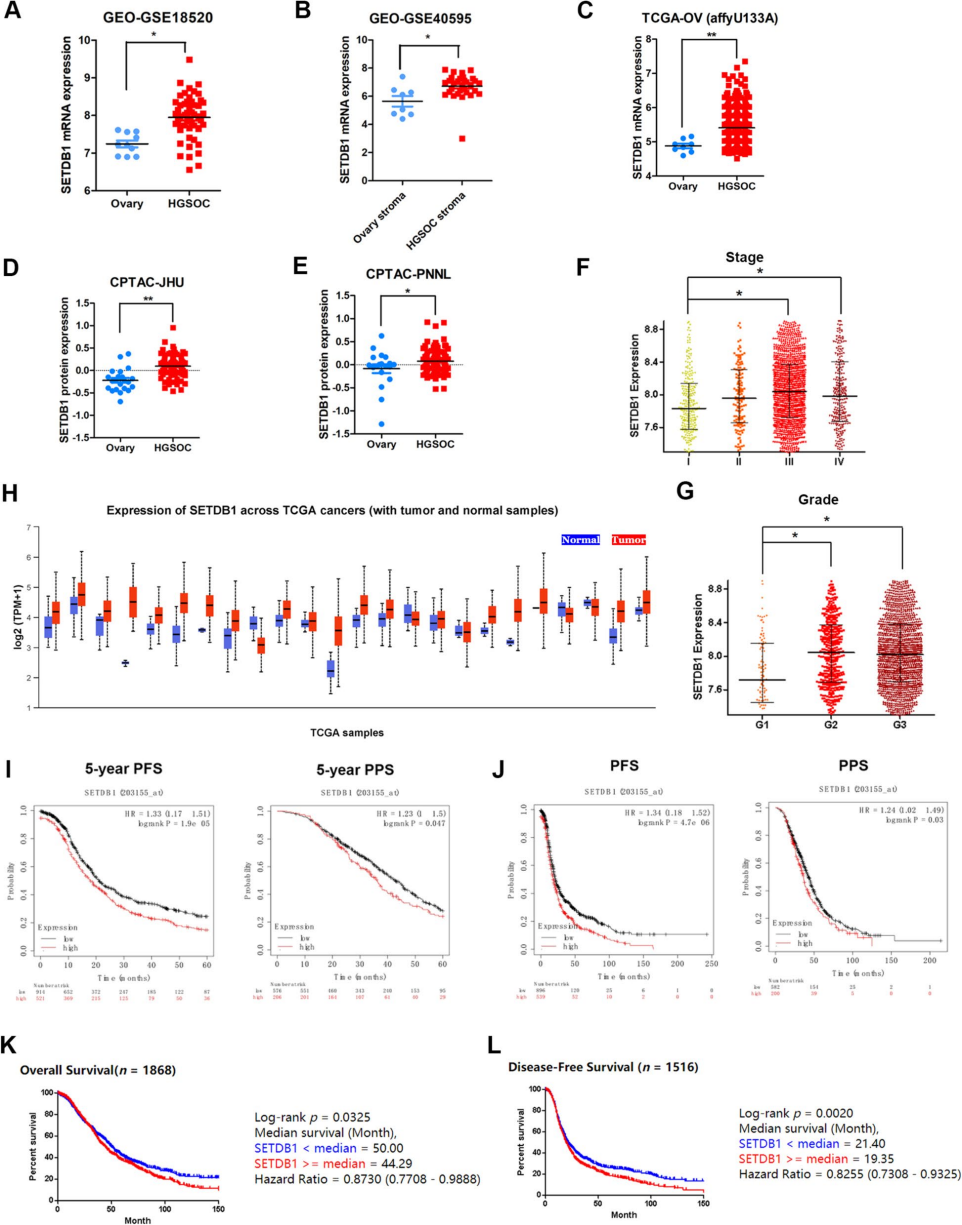
SETDB1 is highly expressed in ovarian cancer and associated with poor prognosis. **A** SETDB1 mRNA expression in ovarian cancer and normal ovarian epithelial tissue from GSE18520. **B** SETDB1 mRNA expression in ovarian cancer stroma and normal ovary stroma from GSE40595. **C** SETDB1 mRNA expression in ovarian cancer and normal ovarian epithelial tissue from TCGA database. **D**, **E** SETDB1 protein expression in ovarian cancer and normal ovary tissue from CPTAC. **F**, **G** Expression of SETDB1 in different stages and grades of ovarian cancer from CSIOVDB database. **H** Expression of SETDB1 in pan-cancer. **I**, **J** Kaplan–Meier analysis showed the effect of SETDB1 expression on PFS or PPS of ovarian cancer patients from K-M plotter. **K**, **L** Kaplan–Meier analysis showed the effect of SETDB1 expression on OS or PFS of ovarian cancer patients from CSIOVDB

To explore the expression of SETDB1 in OC, we analysed SETDB1 mRNA expression from two GEO datasets (GSE18520 and GSE40595) and one TCGA dataset (affyU133a). The results of the analyses showed that the histone lysine methyltransferase SETDB1 was overexpressed in OC tissue compared with normal ovarian tissue in the two GEO databases (Fig. 1A and B) and TCGA dataset (Fig. 1C). Similarly, the SETDB1 protein expression in OC tissue was also significantly increased than that in normal ovarian tissue (Fig. 1D and E). Importantly, the expression of SETDB1 was related to clinical stage and grade in ovarian cancer patients (Fig. 1F and G). Interestingly, SETDB1 was overexpressed in various cancers (Fig. 1H).

Next, we analysed the correlation between SETDB1 expression and the prognosis of OC patients through public databases. Kaplan‒Meier analysis showed that the higher expression of SETDB1 predicted poor progression-free survival (PFS) and poor post-progression survival (PPS) in ovarian cancer patients (Fig. 1I and J). The same results showed that higher expression of SETDB1 was correlated not only with worse PFS but also with poor overall survival (OS) from CSIOVDB (Fig. 1K and L).

### SETDB1 promotes ovarian cancer cell proliferation in vitro

**Fig. 2:**
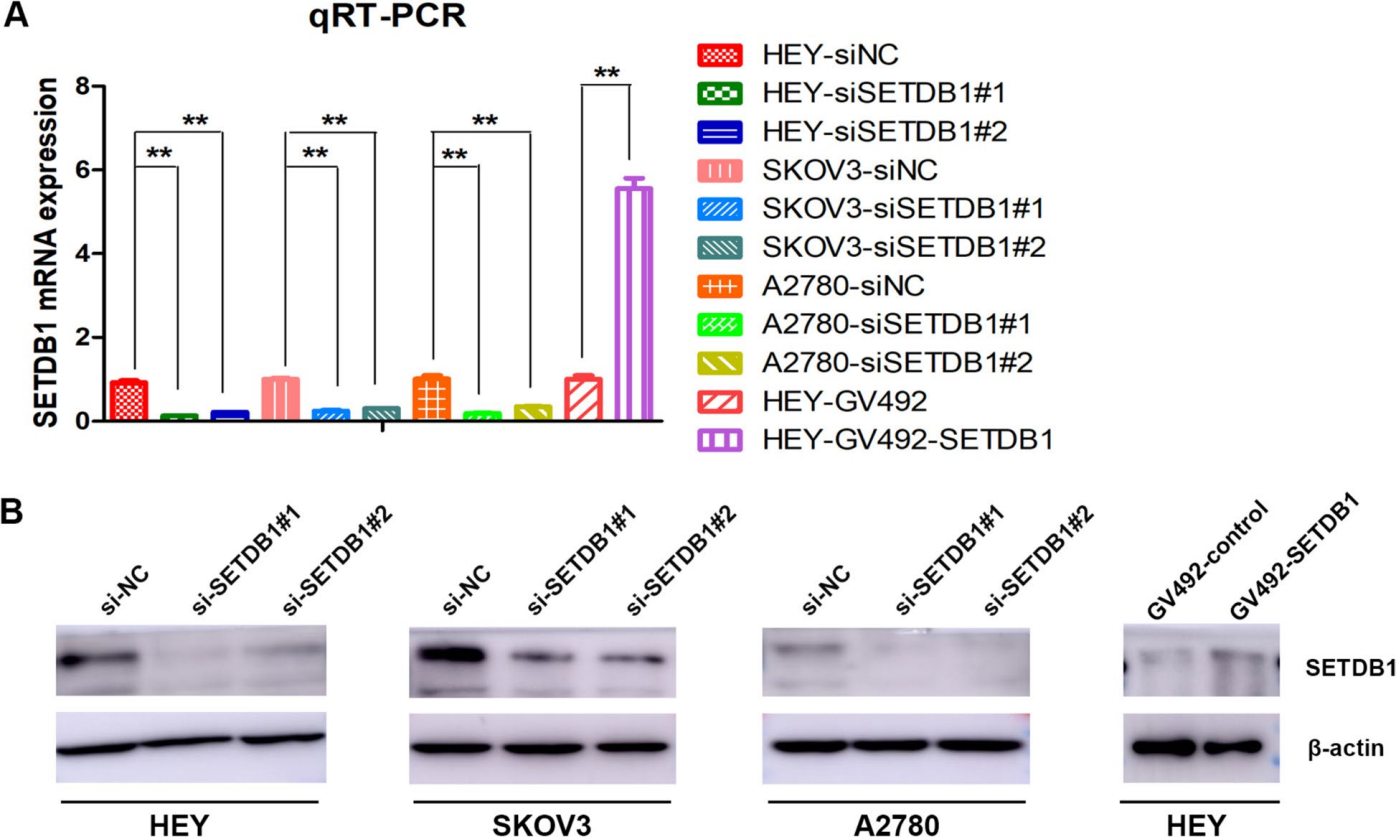
Overexpression and knockdown efficiency of SETDB1. **A** The efficiency of SF3B4 mRNA overexpression and knockdown was detected by qRT-PCR. **B** Up- and down-regulation efficiency of SETDB1 protein was confirmed by WB

To explore the effect of SETDB1 in ovarian cancer progression, SETDB1 knockdown and overexpression cell lines were constructed. First, we used SETDB1 siRNA to transiently transfect HEY, SKOV3 and A2780 cells to knockdown SETDB1. Next, we constructed SETDB1 stable overexpressing HEY cell lines via lentivirus infection. The SETDB1 mRNA and protein expression were obviously downregulated after SETDB1 knockdown. On the contrary, the expression of SETDB1 mRNA and protein increased remarkably after SETDB1 overexpression (Fig. 2A and B).

**Fig. 3:**
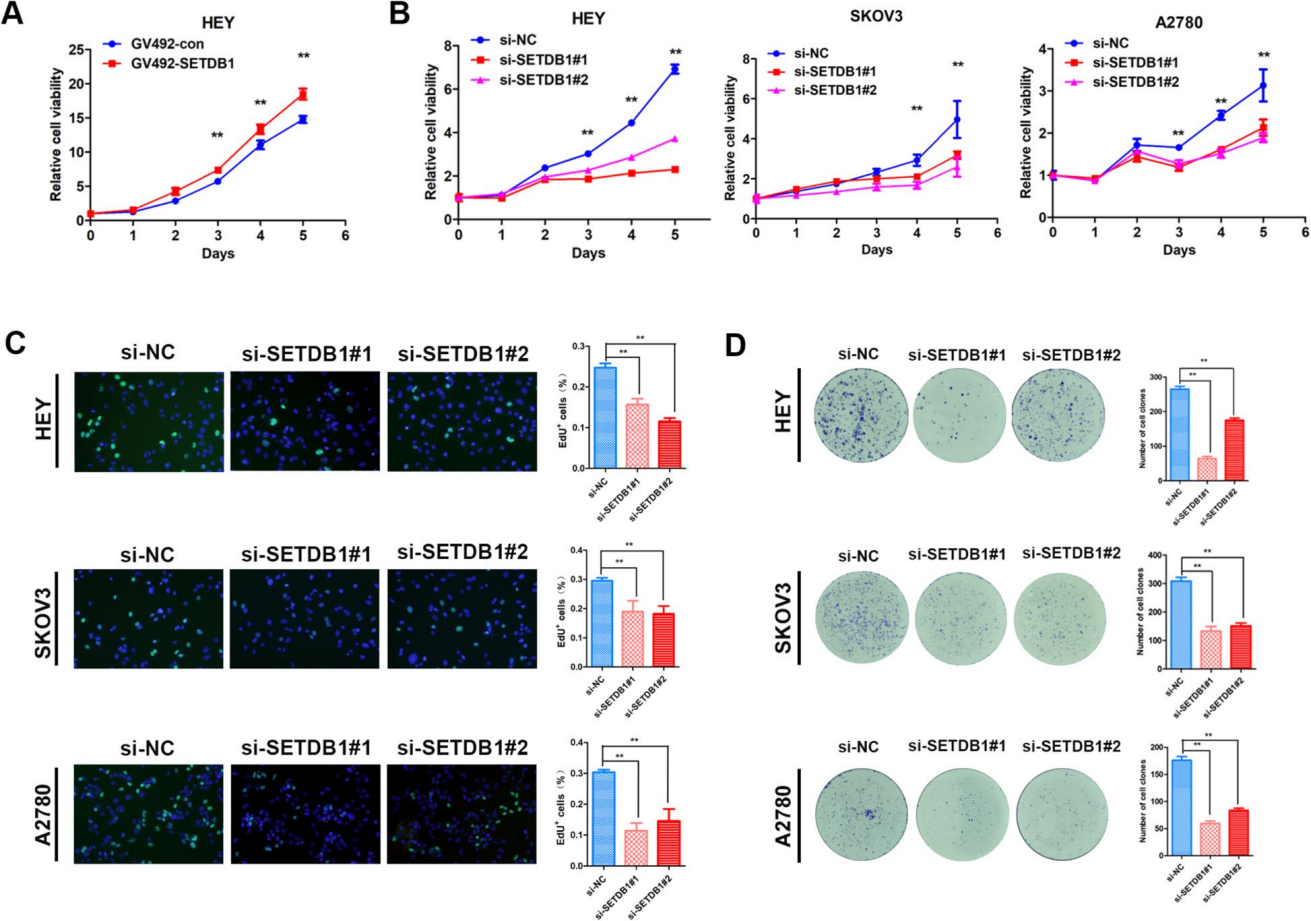
SETDB1 promotes ovarian cancer cells proliferation in vitro. **A**, **B** MTT assay was performed to detect the impact of SETDB1 overexpression and knockdown on growth ability in ovarian cancer cells. **C** EdU assay was performed to analyze the proliferation of ovarian cancer cells upon SETDB1 knockdown. **D** Colony formation assay was performed to evaluate the effect of SETDB1 on clonality in ovarian cancer cells

We performed the MTT and EdU assays to determine whether SETDB1 can regulate the proliferation of ovarian cancer cells. The results revealed that upregulation of SETDB1 expression promoted the proliferation of HEY cells (Fig. 3A). In contrast, downregulation of SETDB1 inhibited the proliferative ability of HEY, A2780 and SKOV3 cells (Fig. 3B and C). The colony formation experiment suggested that the clonality of HEY, SKOV3 and A2780 cells obviously decreased after silencing SETDB1 (Fig. 3D).

### Knockdown of SETDB1 suppresses invasion and metastasis of ovarian cancer cells

**Fig. 4:**
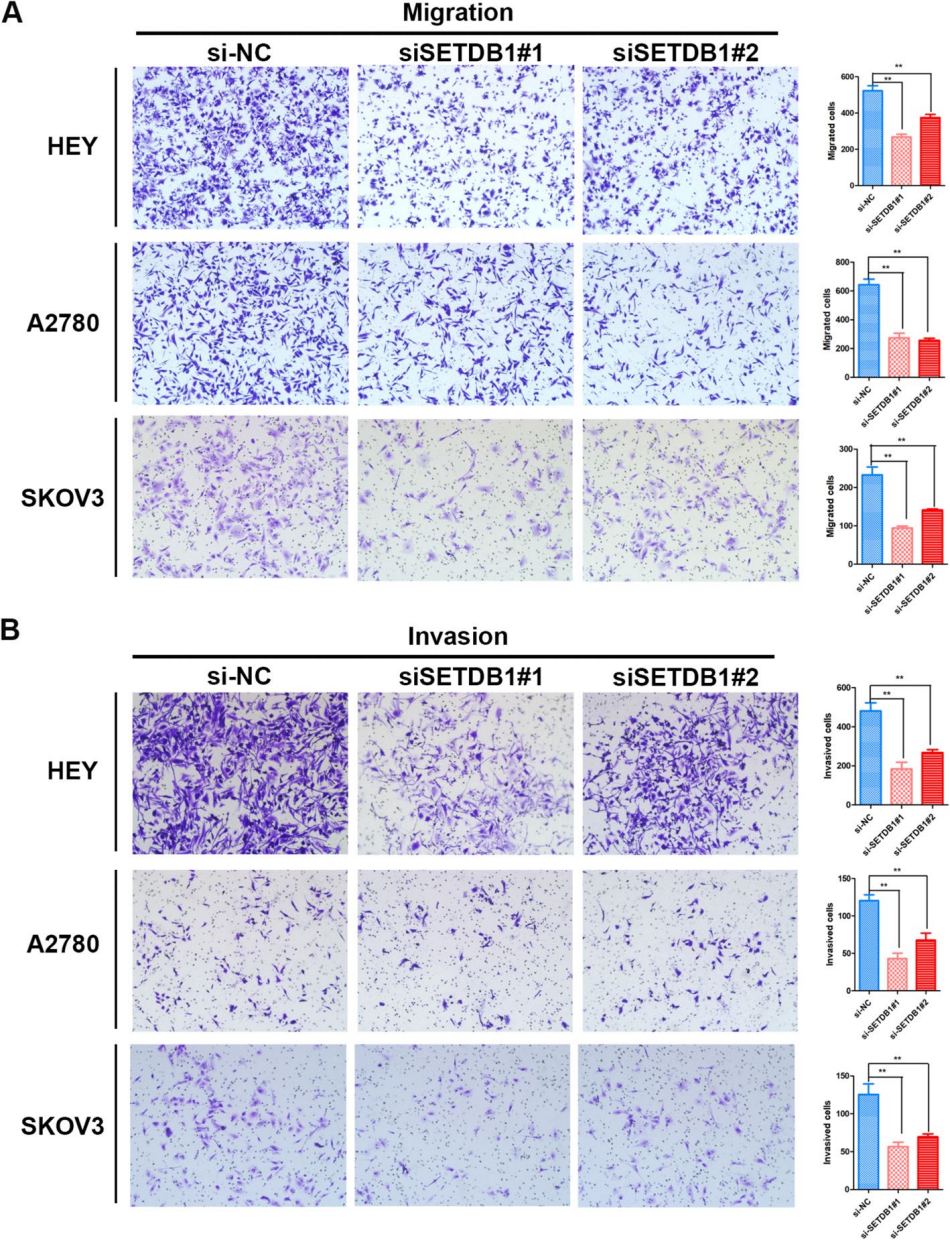
SETDB1 promotes ovarian cancer cells mobility in vitro. **A** Transwell assay showed the effect of SETDB1 on migration ability in ovarian cancer cells. **B** The effect of invasion ability of HEY, A2780 and SKOV3 cells was showed after SETDB1 downregulated

Subsequently, we assessed the effect of SETDB1 during ovarian cancer metastasis. In our experiment, results of the cell migration assay indicated that the number of migrated cells was obviously reduced in the siSETDB1 group than in the control group (Fig. 4A). The same results were observed in the cell invasion assay (Fig. 4B). Therefore, our results confirmed that SETDB1 promotes the migration and invasion of ovarian cancer cells.

### SETDB1 promotes the transcription of SF3B4

**Fig. 5:**
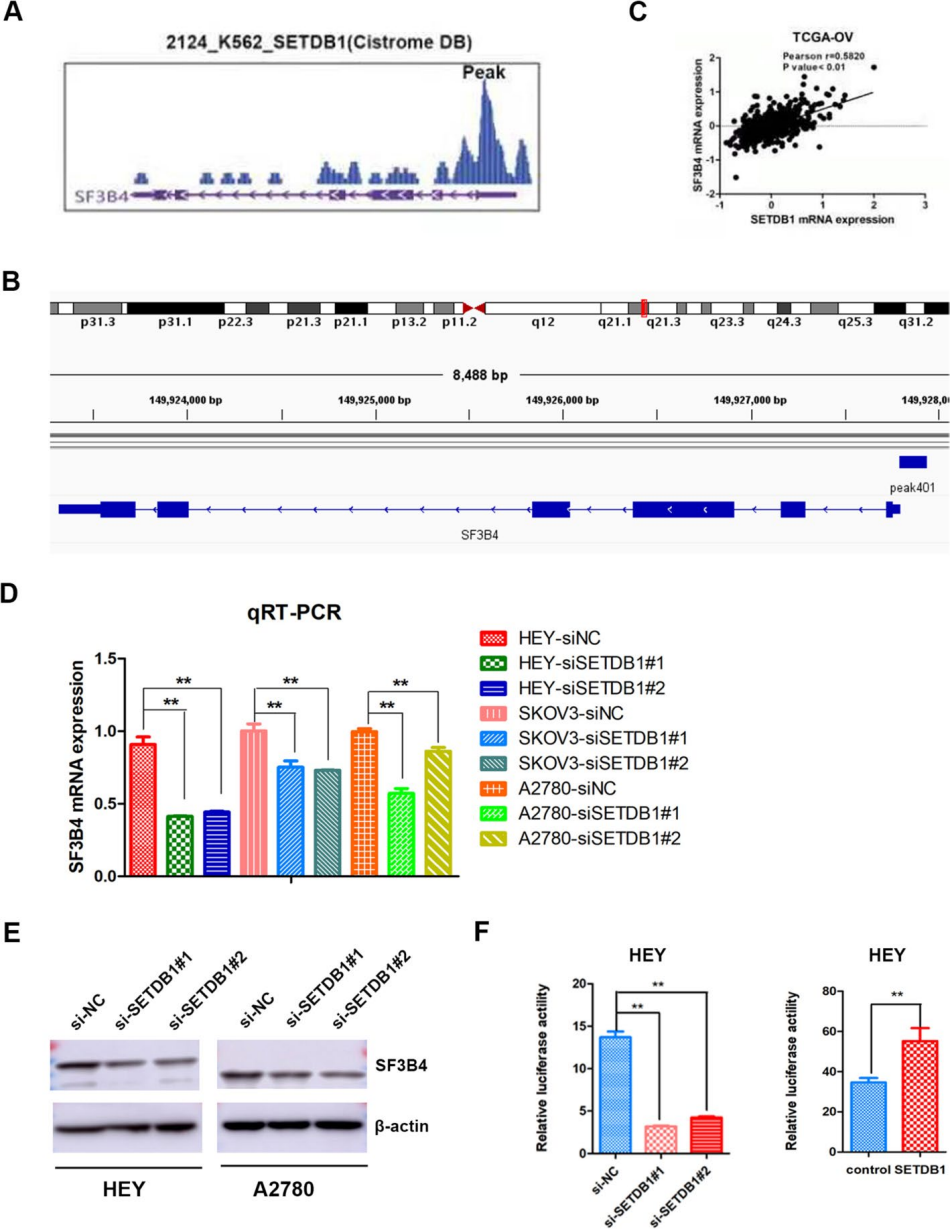
SETDB1 promotes the expression of SF3B4 in ovarian cancer. **A** Cistrome DB data showed the binding peak of SETDB1 protein in the SF3B4 promoter region. **B** Visualization analysis of Cistrome project 2124 data by IGV showed the SETDB1 binding site in SF3B4 promoter. **C** TCGA-OV database showed the positive correlation between SETDB1 and SF3B4 mRNA expression in ovarian cancer. **D**, **E** qRT-PCR and WB showed the effect of SETDB1 knockdown on SF3B4 mRNA and protein expression in ovarian cancer. **F** Luciferase reporter assays showed the effect of SETDB1 on SF3B4 promoter in HEY cells

Although SETDB1 has been reported to play an important role in several tumours, the mechanism by which SETDB1 regulates ovarian cancer progression remains unclear. SETDB1 usually acts as a methyltransferase to silence gene expression, and can also act as a transcription factor to activate downstream gene expression. SF3B4 is a core splicing factor and our research showed that SF3B4 promotes ovarian cancer progression by improving the splicing efficiency of RAD52 [38]. Interestingly, we found a SETDB1 binding site in the promoter of the SF3B4 gene through the Cistrome DB database (Fig. 5A and B). The correlation analysis between SETDB1 and SF3B4 mRNA expression showed that their expression was positively correlated (Fig. 5C). Therefore, we hypothesized that SETDB1 might act as a transcription factor to activate SF3B4 expression.

To explore the effect of SETDB1 knockdown on SF3B4 expression, qRT‒PCR and WB was performed. The results revealed that the expression of SF3B4 mRNA decreased notably after SETDB1 knockdown in ovarian cancer cells (Fig. 5D). The same result was observed in WB experiment (Fig. 5E).

To further confirm whether SETDB1 can bind to the promoter of SF3B4 and promote SF3B4 expression, we cloned a full-length fragment of the SF3B4 promoter into the pGL 4.26 vector, and then, a dual-luciferase reporter gene assay was performed. SETDB1 overexpression obviously increased the luciferase activity of SF3B4 promoter, whereas SETDB1 knockdown notably decreased the luciferase activity of SF3B4 promoter (Fig. 5F). The above results implied that SETDB1 can bind to SF3B4 promoter.

### SF3B4 deficiency impaired the biological effects of SETDB1 overexpression

Our previous research has shown that down-regulation of SF3B4 suppresses the proliferative ability and mobility of ovarian cancer cells [38]. Therefore, we performed rescue experiments to investigate whether SF3B4 deficiency can weaken the biological effects of SETDB1 upregulation.

**Fig. 6:**
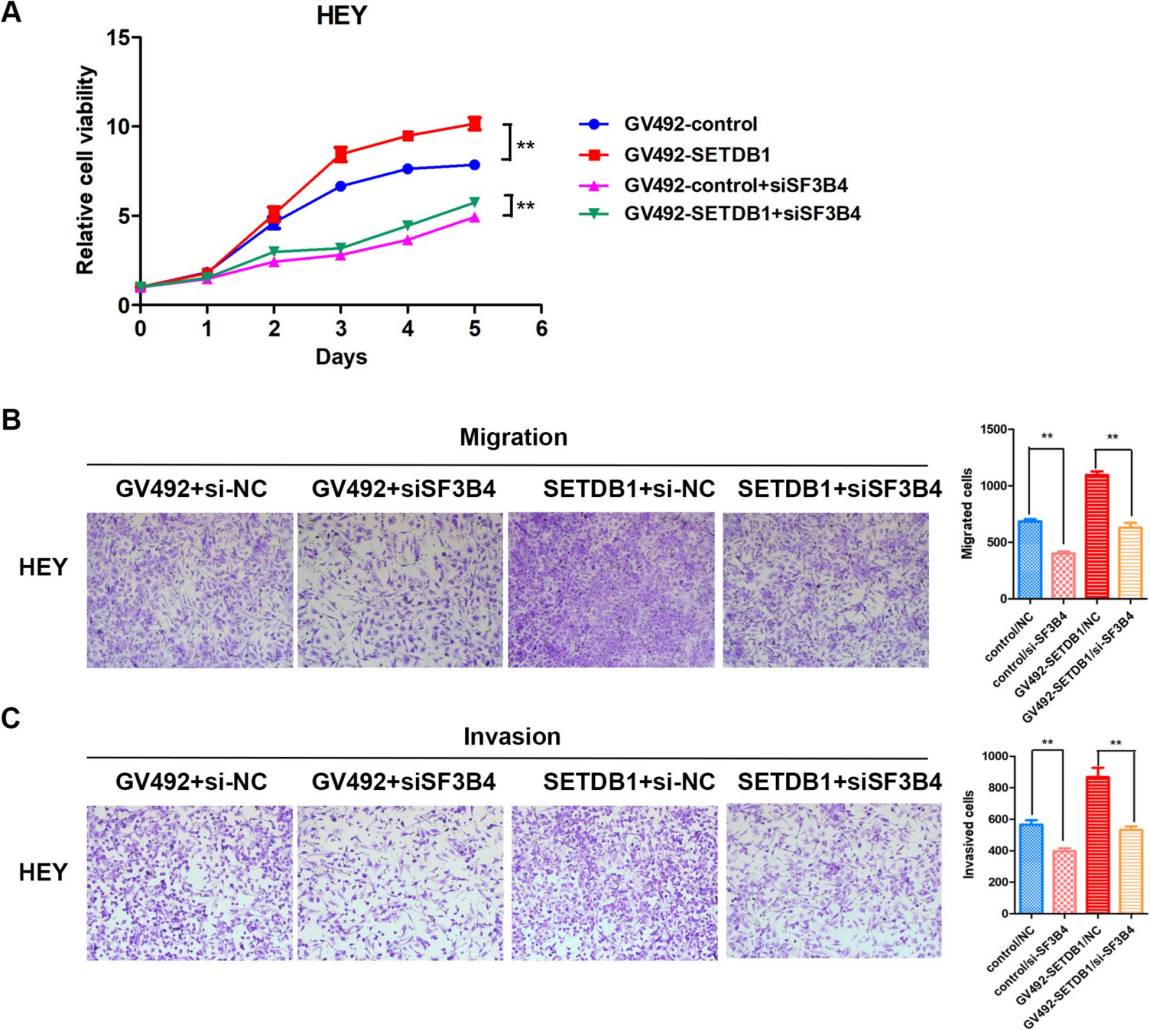
Knockdown of SF3B4 weakens the biological effects of SETDB1 overexpression in ovarian cancer cells. **A** MTT assay detected the effect of SETDB1 and SF3B4 different expression on proliferation in HEY cells. **B**, **C** Transwell assays showed the effect on migration and invasion ability of SF3B4 deficiency on SETDB1 stable-overexpressed HEY cells

We constructed SETDB1 stably overexpressing HEY cell lines and knocked down SF3B4 expression in them. The results of the MTT assay showed that down-regulation of SF3B4 weakened the proliferation-promoting effect of SETDB1 (Fig. 6A). Similarly, SF3B4 knockdown also impaired the effect of SETDB1 to promote the mobility of ovarian cancer cells (Fig. 6B and C).

### SETDB1 regulates the stromal microenvironment of ovarian cancer

Many tumours have abnormal immune function. Tumours evade immune detection through multiple methods. Studies have shown that epigenetic dysregulation is related to immune escape. SETDB1 is a H3K9 methyltransferase and is associated with immune cell function and tumour immunity [22, 23].

**Fig. 7:**
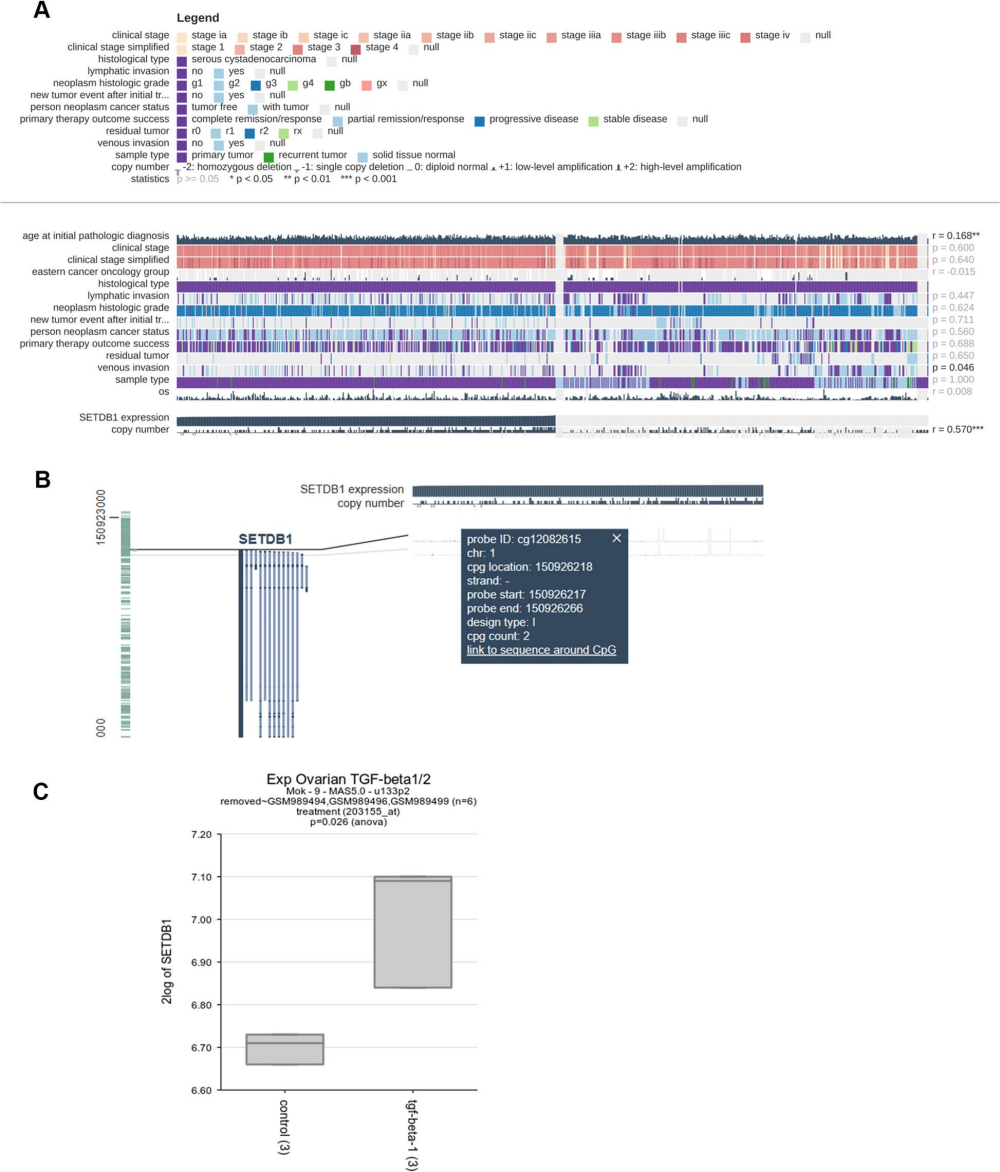
SETDB1 methylation is amplified in ovarian cancer. **A** SETDB1 methylation amplification was found through MEXPRESS database in ovarian cancer. **B** The site of SETDB1 methylation amplification in ovarian cancer. **C** Relative SETDB1 expression upon TGF-β treatment

We found an amplification of the SETDB1 methylation site in ovarian cancer through a public database (Fig. 7A and B). Moreover, the amplification of this site was obviously associated with venous invasion (Fig. 7A). In addition, we found that SETDB1 expression was obviously increased in ovarian fibroblasts treated with TGF-β than in the control group (Fig. 7C). Therefore, we speculate that SETDB1 may be involved in vascular remodeling in ovarian cancer and associated with epithelial-mesenchymal transformation.

**Fig. 8:**
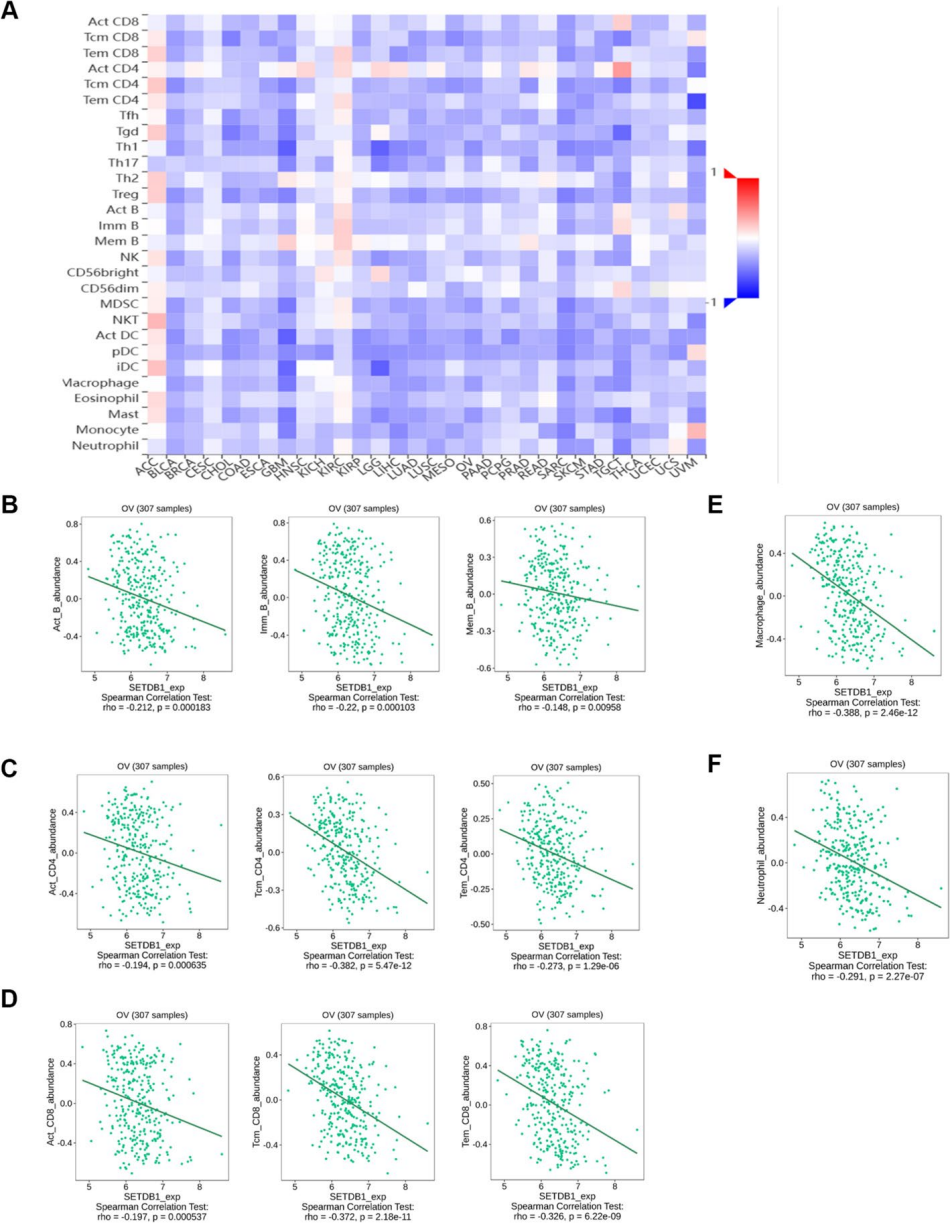
Overexpression of SETDB1 impairs the function of immune cells. **A** Heatmap showed correlation analysis between SETDB1 and immune cells in pan-cancer. **B**-**F** Correlation analysis between SETDB1 and abundance of B cells, CD4^+^ T cells, CD8^+^ T cells, macrophage and neutrophil in ovarian cancer

The results of bioinformatics analysis revealed that SETDB1 affects the function of immune cells in many diseases (Fig. 8A). In ovarian cancer, the increased expression of SETDB1 was accompanied by a decreased abundance of multiple immune cells. These cells include B cells (Fig. 8B), CD4 + T cells (Fig. 8C), CD8 + T cells (Fig. 8D), macrophages (Fig. 8E), and neutrophils (Fig. 8F). Therefore, SETDB1 inhibits tumour immunity in OC, and the function of SETDB1 in the immune system of OC needs to be further elucidated.

## Discussion

Epigenetic regulation causes alteration of gene activity independent of changes in DNA sequence, such as histone modifications and DNA methylation [40]. Epigenetic dysregulation is a common event in tumorigenesis [9]. Histone modification is a common epigenetic modification, and histone methylation is an important histone modification method [41]. SETDB1 was discovered in 1999 [8], and identified as a H3K9 methyltransferase in 2002 [42, 43]. Its main physiological function is to mediate methylation of histone proteins in chromatin, leading to gene silencing. In addition, SETDB1 is involved in disease regulation by promoting AKT kinase activity through AKT methylation [20, 21]. In the recent years, SETDB1 has been reported to be participated in the regulation of immune function [14, 22, 23, 44–48].

Researches have shown that SETDB1 is upregulated in majority of cancers and promotes cancer malignant biological behavior [49]. Less commonly, SETDB1 inhibits tumour progression, depending on the stage and type of the tumour [49]. The TCGA database shows that the SETDB1 gene is amplified in many tumours, such as liver cancer, bladder cancer, breast cancer, uterine cancer and melanomas, and in ovarian cancer, 7.4% of the SETDB1 gene is amplified [50, 51]. In tumorigenesis, SETDB1 downregulates the expression of tumour suppressor factors in most cases. For example, in colorectal cancer (CRC), SETDB1 promotes CRC development by epigenetically silencing p21 expression [13]. In a mouse model of pancreatic cancer, SETDB1 weakens p53-mediated apoptosis and affects the progression of pancreatic cancer [16]. SETDB1 regulates the growth of liver cancer cells via methylation of p53 [17].

In previous studies, circSETDB1 was shown to promote ovarian cancer progression and act as a biomarker for prognosis and drug resistance [27, 28]. However, the effect of SETDB1 protein in ovarian cancer remains unclear. In our study, we found that SETDB1 was over-expression in ovarian cancer, and its upregulation was related to poor PFS, OS and PPS of OC patients. Then, we verified that knockdown of SETDB1 inhibited the proliferative ability and invasion of OC cells. In contrast, SETDB1 upregulation promoted OC cell progression. These findings are consistent with most other studies [7, 10, 19, 52, 53]. Therefore, SETDB1 plays a cancer-promoting effect in ovarian cancer.

In Drosophila melanogaster, SETDB1 can either inhibit or promote gene expression, and this phenomenon is related to its binding position in chromatin [54]. Similarly, in human cells, SETDB1 can not only silence gene expression through the activity of methyltransferase but also promote gene expression through the activity of transcription factor. For example, SETDB1 can interact with Tiam1 to promote its expression, and further promote the progression of liver cancer [24]. In breast cancer, SETDB1 binds to Snail promoter and increases its expression and then promoting the EMT of MCF7 cells [18]. SETDB1 upregulates STAT1 expression via binding to its promoter, promoting CRC progression through the STAT1-CCND1/CDK6 axis [25]. SETDB1 mediates the malignant biological behaviour of gastric cancer by interacting with ERG and enhancing the promoter activity of CCND1 and MMP9 [7]. In conclusion, SETDB1 can act as a transcription factor and promote gene expression in tumours.

In our study, we revealed that SETDB1 can interact with SF3B4 promoter through bioinformatics analysis. Then, we proved that SETDB1 protein could bind and activate the promoter of SF3B4 and increase its transcription and expression through a dual-luciferase reporter assay. SF3B4 mRNA and protein expression level decreased obviously after SETDB1 knockdown. Rescue experiments showed that SF3B4 knockdown obviously weakened the promoting effect of SETDB1 overexpression on the malignant behaviour of OC cells. Therefore, SETDB1 promotes OC progression by positively regulating SF3B4.

Studies have shown that epigenetic dysregulation is a common feature in tumours, and is involved in immune escape [55, 56]. SETDB1 suppresses the intrinsic immunogenicity of tumours through epigenetic silencing and represents a candidate target for immunotherapy [23]. In cancer cells, upregulation of SETDB1 inhibits the production and infiltration of antitumour immune cells, interferes the expression of PD-L1, disrupts type I interferon response, and IFN signaling [22]. In melanoma, KDM5B recruits SETDB1 to repress its targets and further promotes immune evasion [45]. In a murine lung adenocarcinoma model and murine skin melanoma model, SETDB1 inhibits endogenous retrovirus expression and the type I interferon response and restrains antitumour immunity during radiotherapy, suggesting that downregulation of SETDB1 may be a promising way to enhance the efficacy of radiotherapy [14]. Similarly, in ovarian cancer, the SETDB1-TRIM28 complex represses the expression of PD-L1 and the infiltration of CD8^+^ T cells and inhibits antitumour immunity by regulating the cGAS-STING pathway [44]. In this study, we found an amplification of the SETDB1 methylation site in ovarian cancer, and the upregulation of SETDB1 decreased the expression of multiple immune cells. The effect of SETDB1 on immunity in OC deserves further exploration.

In summary, our study verified that SETDB1 was up-regulated and associated with poor PFS, OS and PPS in ovarian cancer patients. SETDB1 promoted OC progression by promoting SF3B4 expression. Moreover, SETDB1 was involved in regulating the immune microenvironment in ovarian cancer. SETDB1 may be a promising target for OC therapy.

Although we validated that SETDB1 promoted ovarian cancer progression by promoting the transcription of SF3B4, our study still has several limitations. First, due to the complexity of molecular regulatory mechanisms, SETDB1-centered regulatory network needs further experimental confirmation not only in vitro, but also in vivo experiments. Furthermore, SETDB1 has been shown to be associated with drug resistance in cancers. It is well known that recurrence and drug resistance are the difficulties in ovarian treatment, whether SETDB1 is involved in chemotherapy resistance of ovarian cancer and its mechanism need to be further explored.

## Conclusion

Through the current study, we can draw the conclusions that SETDB1 is a risk factor for prognosis in ovarian cancer patients. Its high expression can transcriptionally activate SF3B4 and then promote the proliferation and metastasis of ovarian cancer. The tumor microenvironment includes many types of cells and molecules such as tumor cells, immune cells, fibroblasts and extracellular matrix. They form complex networks that play a role in tumor progression. We found SETDB1 may regulate the microenvironment of ovarian cancer and affect epithelial-mesenchymal transformation and immunity through bioinformatics analysis. The mechanism by which SETDB1 affects the microenvironment of ovarian cancer needs to be further studied.

## Data Availability

The datasets used and/or analyzed during the current study are available from the corresponding author on reasonable request.
